# A territory‐wide study on the factors associated with recurrent asthma exacerbations requiring hospitalization in Hong Kong

**DOI:** 10.1002/iid3.419

**Published:** 2021-03-03

**Authors:** Ka Pang Chan, Fanny Wai San Ko, Kwun Cheung Ling, Pik Shan Cheung, Lee Veronica Chan, Yu Hong Chan, Yi Tat Lo, Chun Kong Ng, Macy Mei‐sze Lui, Kwok Sang Wilson Yee, Cee Zhung Steven Tseng, Pak Yiu Tse, Mo Lin Maureen Wong, Kah Lin Choo, Wai Kei Lam, Chun Man Wong, Sheng Sheng Ho, Chung Tat Lun, Christopher Kei Wai Lai

**Affiliations:** ^1^ Department of Medicine & Therapeutics Chinese University of Hong Kong Hong Kong China; ^2^ Department of Medicine & Therapeutics Prince of Wales Hospital Hong Kong China; ^3^ Department of Medicine & Geriatrics United Christian Hospital Hong Kong China; ^4^ Department of Medicine & Geriatrics Princess Margaret Hospital Hong Kong China; ^5^ Department of Medicine Pamela Youde Nethersole Eastern Hospital Hong Kong China; ^6^ Department of Medicine Queen Elizabeth Hospital Hong Kong China; ^7^ Department of Medicine The University of Hong Kong Hong Kong China; ^8^ Department of Medicine Queen Mary Hospital Hong Kong China; ^9^ Department of Medicine & Geriatrics Kwong Wah Hospital Hong Kong China; ^10^ Department of Medicine Tseung Kwan O Hospital Hong Kong China; ^11^ Department of Medicine & Geriatrics Caritas Medical Centre Hong Kong China; ^12^ Department of Medicine North District Hospital Hong Kong China; ^13^ Department of Medicine Alice Ho Miu Ling Nethersole Hospital Hong Kong China

**Keywords:** asthma (clinical aspects), asthma clinical care, asthma control, asthma exacerbations, asthma outcomes research

## Abstract

**Background:**

The real‐world relationships between the demographic and clinical characteristics of asthma patients, their prehospitalization management and the frequency of hospitalization due to asthma exacerbation is poorly established.

**Objective:**

To determine the risk factors of recurrent asthma exacerbations requiring hospitalizations and evaluate the standard of baseline asthma care.

**Methods:**

A territory‐wide, multicentre retrospective study in Hong Kong was performed. Medical records of patients aged ≥18 years admitted to 11 acute general hospitals from January 1 to December 31, 2016 for asthma exacerbations were reviewed.

**Results:**

There were 2280 patients with 3154 admissions (36.7% male, median age 66.0 [interquartile range: 48.0–81.0] years, 519 had ≥2 admissions). Among them, 1830 (80.3%) had at least one asthma‐associated comorbidity, 1060 (46.5%) and 885 (38.9%) of patients had Accident and Emergency Department (AED) attendance and hospitalization in the preceding year, respectively. Patients with advancing age (incidence rate ratio [IRR]: 1.003 for every year increment), a history of AED visits or hospitalization (IRR: 1.018 and 1.070 for every additional episode, respectively) for asthma exacerbation in the preceding year, the presence of neuropsychiatric (IRR: 1.142) and gastrointestinal (IRR: 1.154) comorbidities were risk factors for an increasing number of admissions for asthma exacerbation. For patients with ≥2 admissions, 17.1% were not prescribed inhaled corticosteroid and only 44.6% had spirometry checked before the index admission. Asthma phenotyping was often incomplete, as assessment of atopy (total serum immunoglobulin E level and senitization to aeroallergens) was only performed in 30 (5.8%) patients with ≥2 admissions.

**Conclusions and Clinical Relevance:**

Improving asthma care, especially in elderly patients with a prior history of urgent healthcare utilization and comorbidities, may help reduce healthcare burden. Suboptimal management before the index admission was common in patients hospitalized for asthma exacerbations. Early identification of patients at risk and enhancement of baseline asthma management may help to prevent recurrent asthma exacerbation and subsequent hospitalization.

**Abbreviations**
AED: Accident and Emergency Department
BD: bronchodilator
COPD: chronic obstructive pulmonary disease
FEV1: forced expiratory volume in 1 s
FVC: forced vital capacity
GINA: Global Initiative for Asthma
GOPC: general outpatient clinic
ICS: inhaled corticosteroids
ICU: intensive care unit
IgE: immunoglobulin E
IMV: invasive mechanical ventilation
IQR: interquartile range
IRR: incidence rate ratio
MA: multiple admissions
PEFR: peak expiratory flow rate
RAST: radioallergosorbent test
SA: single admission
SABA: short‐acting beta‐agonist
SAMA: short‐acting muscarinic antagonist

## 1 INTRODUCTION

Asthma is the most prevalent chronic respiratory disease worldwide.^1^, ^2^ The global prevalence of asthma is 4.3%,^3^, ^4^ ranging from 12.6% in schoolchildren to 8.1% in the elderly.^2^, ^5^ A recent Chinese study reported that 7.2% of patients had at least one hospital admission due to asthma exacerbation in the preceding year.^3^ Severe exacerbations, especially those requiring hospitalization, are associated with significantly more morbidities (decline in lung function and quality of life),^6^, ^7^ mortality^8^ and healthcare costs.^9^, ^10^ Therefore, prevention of asthma exacerbations and subsequent hospitalizations are the crucial goals of asthma therapy. Various factors associated with recurrent asthma exacerbations, including asthma‐related comorbidities,^11^ recent asthma exacerbation,^12^ reduced spirometric parameters^13^ and high blood eosinophil counts,^14^ have been reported. However, the association between these factors, level of asthma control, baseline asthma workup, follow‐up status and the burden of recurrent asthma exacerbations requiring hospitalization remains to be established. This study aimed at evaluating the clinical characteristics of patients who had been hospitalized for asthma exacerbations in the past 12 months and exploring the risk factors that were associated with multiple admissions.

## 2 METHODS

This was a territory‐wide, multicentre retrospective study in Hong Kong. Eleven acute general public hospitals participated in this study.

### 2.1 Case recruitment

By using the International Classification of Diseases, Ninth Revision, Clinical Modification code 493.xx, each participating hospital identified all admissions to the medical department with a primary hospital discharge diagnosis of asthma exacerbation over 12 months (January 1 to December 31, 2016). Asthma exacerbation was defined as an event characterized by a worsening of asthma control status requiring the use of systemic corticosteroids (OCS), or an increase from a stable maintenance dose, for at least 3 days.^15^

All relevant electronic and paper medical records of the included admissions were reviewed manually by respiratory specialists of the corresponding hospital. Patients who were ≥18 years old on the day of admission were included in the analysis. Patients with an inappropriate diagnosis of asthma exacerbation or incomplete drug records were excluded. Patients admitted to different hospitals were cross‐referenced to verify the exact number of admissions in the Year 2016.

### 2.2 Assessment of subjects

Clinical information of the patients including baseline demographics, asthma‐related comorbidities, asthma history and related care including a history of prior severe asthma exacerbation requiring intensive care unit (ICU) admission and invasive mechanical ventilation (IMV), use of urgent healthcare utilization due to asthma exacerbation in the preceding year, assessment of symptom control (daytime symptoms, night waking, short‐acting beta‐agonist [SABA] reliever use and activity limitation due to asthma in the past 4 weeks based on the Global Initiative for Asthma [GINA] recommendation in the last clinic visit),^16^ previous workup on asthma (spirometry, measurement of peak expiratory flow rate [PEFR], blood eosinophils count, total serum immunoglobulin E [IgE] level, allergy skin prick tests, and radioallergosorbent test [RAST]), pharmacological treatment before the index admission and follow‐up status were collected.

Comparisons were performed between patients with single admission (SA; one hospitalization) and multiple admissions (MA, ≥1 hospitalization) for asthma exacerbation in the Year 2016, to identify the patient‐based risk factors for recurrent exacerbations requiring hospitalizations. Comparisons were also performed between patients with and without long‐term follow‐up, to evaluate any difference in the baseline characteristics.

### 2.3 Statistics

Data were analyzed by the Statistical Package of the Social Science Statistical Software (SPSS) for Window, Version 26 (IBM SPSS In.c). The demographic and clinical characteristics were presented with descriptive statistics, as mean with *SD*, median with interquartile range (IQR) and absolute number with percentage as appropriate. Comparison between patient groups was performed by chi‐square or Fisher's exact test for categorical variables, and independent *t* test or Mann–Whitney *U* test for continuous variables. Significance of potential risk factors for asthma exacerbations leading to hospitalization was identified by Poisson regression analysis. All tests were two‐tailed, and a value of *p* < .05 was considered significant.

### 2.4 Ethics approval

Ethics application was approved by the corresponding clinical research ethics committee for each participating hospital, with the name of committees and approval reference numbers listed as follow: Joint Chinese University of Hong Kong‐New Territories East Cluster Clinical Research Ethics Committee (CREC 2017.503), Research Ethics Committee (Kowloon Central/Kowloon East) (KC/KE‐17‐0206/ER‐4, KC/KE‐17‐0218/ER‐4, KC/KE‐17‐0235/ER‐1), Kowloon West Cluster Research Ethics Committee (KW/EX‐17‐148(117‐15)), Hong Kong East Cluster Research Ethics Committee (HKECREC‐2017‐082) and Institutional Review Board of the University of Hong Kong/Hospital Authority Hong Kong West Cluster (UW 17‐507) (Table S1). Patient consent was exempted by the committees as it was a retrospective study and no patient identifiers were obtained. The study was conformed to the standards of the Declaration of Helsinki.

## 3 RESULTS

A total of 3515 admissions were identified. After manual review of records, 361 admissions were excluded due to inappropriate discharge coding (343), incomplete drug records (15) and duplication of patients (3). Data of 3154 hospitalization episodes from 2280 patients remained for analysis.

### 3.1 Baseline characteristics and comorbidities

The baseline demographics, clinical characteristics, healthcare utilization related to asthma exacerbation and drug prescription are listed in Table 1. The cohort had elderly and female predominance. Most patients (1832, 80.3%) had at least one asthma‐associated comorbidity. More than half of them had coexisting metabolic diseases (1214, 53.2%), followed by cardiovascular (1132, 49.6%) and atopic diseases (854, 37.5%). A detailed breakdown of asthma‐associated comorbidities is listed in Table S2.

**Table 1:** Baseline characteristics, use of asthma medications, prior asthma care and workup of the cohort, and comparison between SA and MA patients

	Whole cohort	SA	MA	
	(*n* = 2280)	(*n* = 1761)	(*n* = 519)	*p Value*
Age, years, median (IQR)	66.0 (48.0–81.0)	63.0 (44.0–80.0)	76.0 (59.0–86.0)	<.001
Male, *n* (%)	836 (36.7)	655 (37.2)	181 (34.9)	.335
Chinese, *n* (%)	2164 (94.8)	1660 (94.6)	504 (97.1)	.021
Life‐long nonsmokers, *n* (%)	1478 (64.8)	1134 (64.4)	344 (66.3)	.429
Active smokers, *n* (%)	285 (12.5)	238 (13.5)	47 (9.1)	.007
Presence of at least 1 comorbidity, *n* (%)	1830 (80.3)	1363 (77.4)	467 (90.2)	<.001
Metabolic comorbidities^a^, *n* (%)	1214 (53.2)	880 (50.0)	334 (64.4)	<.001
Cardiovascular comorbidities^b^, n (%)	1132 (49.6)	811 (46.1)	321 (61.8)	<.001
Atopic comorbidities^c^, *n* (%)	854 (37.5)	663 (37.6)	191 (36.8)	.726
Respiratory comorbidities^d^, *n* (%)	743 (32.6)	562 (31.9)	181 (34.9)	.206
Neuropsychiatric comorbidities^e^, *n* (%)	471 (20.7)	319 (18.1)	152 (29.3)	<.001
Gastrointestinal comorbidities^f^, *n* (%)	201 (8.8)	139 (7.9)	62 (11.9)	.004
Musculoskeletal comorbidities^g^, *n* (%)	199 (8.7)	141 (8.0)	58 (11.2)	.025
Genitourinary comorbidities^h^, *n* (%)	146 (6.4)	109 (6.2)	37 (7.1)	.442
Nil inhaler, *n* (%)	383 (16.8)	350 (19.9)	33 (6.4)	<.001
SABA or SAMA only, *n* (%)	358 (15.7)	319 (18.1)	39 (7.5)	<.001
Nil controller^i^, *n* (%)	782 (34.3)	700 (39.8)	82 (15.8)	<.001
ICS‐containing regimen, *n* (%)	1470 (64.5)	1040 (59.1)	430 (82.9)	<.001
Oral corticosteroid use, *n* (%)	39 (1.7)	20 (1.1)	19 (3.7)	<.001
Biologics use, *n* (%)	1 (0.0)	0 (0.0)	1 (0.2)	.228
GINA Step 1^j^, *n* (%)	782 (34.3)	700 (39.8)	82 (15.8)	<.001
GINA Step 2^j^, *n* (%)	87 (3.8)	73 (4.1)	14 (2.7)	.130
GINA Step 3^j^, *n* (%)	498 (21.8)	389 (22.1)	109 (21.0)	.598
GINA Step 4^j^, *n* (%)	626 (27.5)	437 (24.8)	189 (36.4)	<.001
GINA Step 5^j^, *n* (%)	285 (12.5)	161 (9.1)	124 (23.9)	<.001
Asthma onset age >18 years old^k^, n (%)	1697 (81.5)	1278 (79.5)	419 (88.6)	<.001
ICU admission ever for asthma exacerbation, *n* (%)	162 (7.1)	115 (6.5)	47 (9.1)	.049
Need of mechanical ventilation ever for asthma exacerbation^l^, *n* (%)	137 (6.0)	96 (5.5)	41 (7.9)	.039
Admission for asthma exacerbation in Year 2015, *n* (%)	885 (38.9)	506 (28.8)	379 (73.0)	<.001
Number of admissions for asthma exacerbation in Year 2015, mean ± *SD*	0.8 ± 2.0	0.4 ± 1.0	2.1 ± 3.5	<.001
AED visit for asthma exacerbation in Year 2015, *n* (%)	1060 (46.5)	663 (37.7)	397 (76.5)	<.001
Number of AED visit for asthma exacerbation in Year 2015, mean ± *SD*	1.1 ± 2.9	0.7 ± 1.5	2.7 ± 5.2	<.001
GOPC visit for asthma exacerbation in Year 2015, *n* (%)	340 (15.0)	248 (14.2)	92 (17.8)	.044
Number of GOPC visit for asthma exacerbation in Year 2015, mean ± *SD*	0.3 ± 1.1	0.3 ± 1.0	0.4 ± 1.4	.112
Regular follow‐up for asthma, *n* (%)	1912 (83.9)^m^	1427 (81.0)	485 (93.4)	<.001
Asthma follow‐up in public sector, *n* (%)	1785 (78.3)	1310 (74.4)	475 (91.5)	<.001
Asthma follow‐up in private sector, *n* (%)	129 (5.7)	119 (6.8)	10 (1.9)	<.001
Asthma that was either partly controlled or uncontrolled^n^, *n (%)*	616 (60.5)	468 (57.1)	148 (74.0)	<.001
Prior spirometry, *n* (%)	711 (36.0)	505 (33.4)	206 (44.6)	<.001
Pre‐BD FEV_1_, L, mean ± *SD (% predicted)*	1.4 ± 0.7 (68.4 ± 23.4)	1.5 ± 0.7 (69.2 ± 22.8)	1.4 ± 0.7 (66.0 ± 24.8)	.114
Pre‐BD FVC, L, mean ± *SD (% predicted)*	2.2 ± 1.0 (83.3 ± 23.1)	2.3 ± 1.0 (84.4 ± 23.4)	2.2 ± 1.0 (80.6 ± 22.3)	.156
Post‐BD FEV_1_, L, mean ± *SD (% predicted)*	1.6 ± 0.8 (73.0 ± 23.5)	1.6 ± 0.7 (75.1 ± 22.7)	1.4 ± 0.8 (68.4 ± 24.8)	.029
Post‐BD FVC, L, mean ± *SD (% predicted)*	2.4 ± 1.0 (87.3 ± 22.3)	2.4 ± 0.9 (89.6 ± 22.2)	2.2 ± 1.0 (82.0 ± 21.7)	.039
Baseline blood eosinophil checked, *n* (%)	1723 (75.6)	1256 (71.3)	467 (90.0)	<.001
Baseline blood eosinophil count, 10/L, median (IQR)	0.2 (0.1–0.4)	0.2 (0.1–0.4)	0.1 (0.0–0.4)	.003
Baseline blood eosinophil count ≥0.2 × 10/L, *n* (%)	970 (56.3)	739 (58.8)	231 (49.6)	.001
Asthma phenotyping^o^, *n* (%)	60 (2.6)	30 (1.7)	30 (5.8)	<.001
Total serum IgE check, *n* (%)	138 (6.1)	79 (4.5)	59 (11.4)	<.001
Total serum IgE level, kU/L, median (IQR)	177 (66–514)	181 (69–672)	172 (67–387)	.446
Allergy skin prick tests, *n* (%)	26 (1.1)	20 (1.1)	6 (1.2)	.969
RAST, *n* (%)	57 (2.5)	29 (1.6)	28 (5.4)	<.001
Action plan discussed^p^, *n* (%)	230 (10.0)	172 (13.2)	58 (16.8)	.086
PEFR check^q^, *n (%)*	30 (1.8)	23 (1.7)	7 (2.0)	.747

*Note*: Statistically significant (*p* < .05).

Abbreviations: AED, Accident and Emergency Department; BD, bronchodilator; FVC, forced vital capacity; FEV_1_, forced expiratory volume in 1 s; GINA, Global Initiative for Asthma; GOPC, General Outpatient Clinic; ICS, inhaled corticosteroids; ICU, Intensive Care Unit; IgE, immunoglobulin E; IQR, interquartile range; MA, multiple admissions; PEFR, peak expiratory flow rate; RAST:, radioallergosorbent test; SA, single admission; SABA, short‐acting beta‐agonist; SAMA, short‐acting muscarinic antagonist.

^a^ Metabolic comorbidities include hypertension, hyperlipidaemia, diabetes mellitus, obesity, hyperthyroidism, hypothyroidism, osteoporosis.

^b^ Cardiovascular comorbidities include hypertension, congestive heart failure, arrhythmia, coronary artery disease, peripheral vascular disease.

^c^ Atopic comorbidities include eczema, urticaria, drug allergy, food allergy, allergic conjunctivitis, allergic rhinitis, nasal polyposis.

^d^ Respiratory comorbidities include lung cancer, chronic obstructive pulmonary disease, bronchiectasis, pulmonary fibrosis, obstructive sleep apnoea, allergic rhinitis, nasal polyposis, sinusitis.

^e^ Neuropsychiatric comorbidities include stroke (ischemic or hemorrhagic), epilepsy, dementia, depression, anxiety, psychotic disorders.

^f^ Gastrointestinal comorbidities include gastroesophageal reflux disease, fatty liver, chronic viral hepatitis.

^g^ Musculoskeletal comorbidities include osteoporosis, osteoarthritis, rheumatoid arthritis, gout.

^h^ Genitourinary comorbidities include benign prostatic hyperplasia, chronic renal disease.

^i^ Irrespective of the use of SABA or SAMA.

^j^ GINA 2016 recommendation.^16^

^k^ Out of the 2081 records with documentation.

^l^ Out of the 2277 records with documentation.

^m^ Two patients had follow‐ups in both public and private clinics.

^n^ Out of the 1019 records with documentation, classified according to the GINA 2016 recommendation.

^o^ If total serum immunoglobulin E plus either allergy skin prick test or radioallergosorbent test were performed.

^p^ Out of the 1652 records with documentation.

^q^ Out of the 1665 records with documentation.

### 3.2 History and prior care of asthma

Most (1697, 74.5%) patients had the onset of asthma ≥18 years old. A total of 162 (7.1%) and 137 (6.0%) patients required ICU admission and IMV for asthma exacerbations respectively, while 885 (38.9%) patients were hospitalized, and 1060 (46.5%) attended the AED at least once for asthma exacerbation in the preceding year.

For asthma follow‐up, 1785 (78.3%) and 127 (5.6%) patients had that in public and private sectors respectively, with 368 (16.1%) patients either had no regular follow‐up for asthma. Before the index admission, blood eosinophil count was available for most patients (1723, 75.6%), with a median count of 0.2 (IQR: 0.1–0.4) × 10^9^/L. About one‐third of patients (711, 36.0%) had spirometry done before. Total serum IgE was checked in 138 (6.1%) patients. Adequate atopic phenotyping, with total serum IgE plus either allergy skin prick tests or RAST, were performed in 60 (2.6%) patients of the whole cohort and 30 (5.8%) MA patients (Table 1). Other aspects of baseline asthma care (documentation of PEFR and discussion of an action plan), were also uncommon (≤10.0%, Table 1).

### 3.3 Baseline drug prescription

A total of 1470 (64.5%) patients received ICS‐containing regimen for asthma, while 782 (34.3%) patients were receiving Step 1 treatment of GINA in the Year 2016 with no controller medication. Use of long‐term OCS and biologics were both uncommon (Table 1). A detailed breakdown of the treatment regimen was listed in Table S3.

### 3.4 Asthma control before the index admission

Asthma symptoms in the 4 weeks preceding the latest clinic visit were recorded in only 1019 patients. Of these, 616 (60.5%) had either partly controlled or uncontrolled asthma, defined by the GINA 2016 recommendation,^16^ before their index admission. The symptom burden based on the four essential questions of symptom control listed by GINA recommendation was listed in Table 2. Daytime symptoms and frequent SABA reliever use were common among patients.

**Table 2:** Frequency of asthma symptoms in the 4 weeks preceding the last clinic visit before the index admission

Asthma symptoms and control	Number of patients with the symptom present/number of patients with the symptom assessment documented
Daytime symptoms more than twice per week, *n* (%)	481/1001 (48.1)
Night waking due to asthma, *n* (%)	271/947 (28.6)
SABA reliever needed more than twice per week, *n* (%)	429/968 (44.3)
Activity limitation due to asthma, *n* (%)	254/932 (27.3)
Asthma that was either partly controlled or uncontrolled (presence of any of the above symptoms)^a^, *n* (%)	616/1019 (60.5)

Abbreviation: SABA, short‐acting beta‐agonist.

^a^ Classified according to the Global Initiation for Asthma 2016 recommendation.^16^

### 3.5 Comparison between patients with and without long‐term follow‐up

Significant differences in various aspects existed between patients with and without long‐term follow‐up (Table 3). Patients who had no long‐term follow‐up were younger, with more active smokers, less asthma‐associated comorbidities, less utilization of ICS‐containing regimen, higher spirometric parameters and less phenotyping workup. None of them had self‐monitoring of peak expiratory flow rate.

**Table 3:** Comparison between patients with and without long‐term follow‐up for asthma

	Whole cohort (*n* = 2280)	Patients with long term follow‐up on asthma (*n* = 1912)	Patients without long term follow‐up on asthma (n = 368)	*p* Value
Age, years, median (IQR)	66.0 (48.0–81.0)	70.0 (54.0–83.0)	38.0 (29.0–52.0)	<.001
Active smokers, *n* (%)	285 (12.5)	167 (8.7)	118 (32.1)	<.001
Presence of at least 1 comorbidity, *n* (%)	1830 (80.3)	1652 (86.4)	178 (48.4)	<.001
ICS‐containing regimen, *n* (%)	1470 (64.5)	1456 (76.2)	14 (3.8)	<.001
Prior spirometry, *n* (%)	711 (36.0)	680 (40.8)	31 (10.1)	<.001
Post‐BD FEV_1_, L, mean ±* SD* (% predicted)	1.6 ± 0.8 (73.0 ± 23.5)	1.5 ± 0.7 (72.5 ± 23.4)	2.3 ± 0.7 (88.3 ± 23.7)	<.001
Post‐BD FVC, L, mean ± *SD* (% predicted)	2.4 ± 1.0 (87.3 ± 22.3)	2.3 ± 0.9 (86.8 ± 22.4)	3.2 ± 0.8 (99.6 ± 15.8)	.001
Baseline blood eosinophil checked, *n* (%)	1723 (75.6)	1579 (82.6)	144 (39.1)	<.001
IgE check, *n* (%)	138 (6.1)	134 (7.0)	4 (1.1)	<.001
Allergy skin prick tests, *n* (%)	26 (1.1)	24 (1.3)	2 (0.5)	.417
RAST, *n* (%)	57 (2.5)	55 (2.9)	2 (0.5)	.009
Action plan discussed, *n* (%)	230 (13.9)	227 (16.4)	3 (1.1)	<.001
PEFR check, *n* (%)	30 (1.8)	30 (2.1)	0 (0.0)	.010

Abbreviations: BD, bronchodilator; FVC, forced vital capacity; FEV_1_, forced expiratory volume in 1 s; ICS, inhaled corticosteroids; IgE, immunoglobulin E; IQR, interquartile range; PEFR, peak expiratory flow rate; RAST, radioallergosorbent test.

### 3.6 Comparison between patients with single admission and multiple admissions

**Figure 1:**
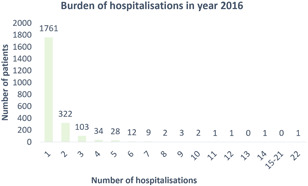
Number of hospitalizations for asthma exacerbation per patient in the Year 2016. The frequency of hospitalizations for asthma patients in the Year 2016 follows a Poisson distribution pattern. A total of 1761 and 519 patients had single and multiple admissions, respectively

**Figure 2:**
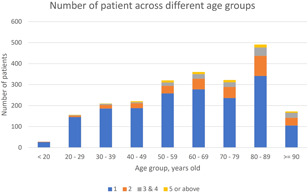
Number of patients hospitalized for asthma exacerbation across different age groups. Hospitalizations for asthma exacerbations were more frequent in patients with advancing age

For asthma exacerbations, 1761 and 519 patients had SA and MA in the Year 2016, respectively. Figure 1 shows the frequency of hospitalizations for these patients, which followed a Poisson distribution pattern. Hospitalizations for asthma exacerbations were more frequent in patients with advancing age (Figure 2). MA patients had a median of 2 (IQR: 2–3) admissions. They were older, less likely to be active smokers and more likely to have comorbidities compared to SA patients. More MA patients had an onset of asthma ≥18 years, history of life‐threatening asthma exacerbation, urgent healthcare utilization due to asthma exacerbation in the preceding year, had either partly controlled or uncontrolled asthma, regular follow‐up for asthma, received care in the public sector and more investigations on asthma than SA patients (Table 1). A significant proportion of patients were not prescribed any controller medication, (39.8% and 15.8% amongst SA patients and MA patients, respectively). Nevertheless, MA patients were more likely to be prescribed with an ICS‐containing regimen and received GINA (2016) Steps 4 or 5 treatment regimens than SA patients. The baseline postbronchodilator spirometric findings and median blood eosinophil count of MA patients were both significantly lower than SA patients (Table S4). More MA patients received a check on total serum IgE and adequate atopic phenotyping than SA patients (Table 1). Detailed comparisons between SA and MA patients are shown in Table 1 and Table S4.

### 3.7 Regression analysis of the factors associated with multiple admissions

Clinical factors significantly associated with MA were included in the Poisson regression analysis (Table 4). Factors with incomplete documentation (such as spirometric parameters and asthma control, with only 36.0% and 44.7% subjects with data available, respectively), although with a significant association, were not included in the analysis. Age and a recent history of urgent healthcare utilization were significantly associated with the increasing number of hospitalizations for asthma exacerbation. For every year increment of age from 18 years old, the incidence rate ratio (IRR) for hospitalization was 1.003. For every additional episode of hospitalization or AED visit for asthma exacerbation in the preceding year, the IRRs were 1.070 and 1.018 times, respectively. The presence of neuropsychiatric (IRR: 1.142) and gastrointestinal (IRR: 1.154) diseases were the only significant comorbidities associated with MA after adjustment of other variables. Use of ICS‐containing medication before the index admission was also associated with multiple admissions. Baseline blood eosinophil count ≤0.2 × 10^9^/L was not significantly associated with hospitalizations.

**Table 4:** Clinical factors associated with the increasing number of admissions by Poisson regression analysis

Parameters	Incidence rate ratio (95% confidence interval)	*p Value*
Age	1.003 (1.000–1.006)	.021
Admission for asthma exacerbation in Year 2015	1.070 (1.057–1.083)	<.001
AED visit for asthma exacerbation in Year 2015	1.018 (1.010–1.027)	<.001
Baseline blood eosinophil count ≤0.2 × 10^9^/L	0.976 (0.902–1.057)	.554
Cardiovascular comorbidities	1.046 (0.918–1.193)	.500
Neuropsychiatric comorbidities	1.141 (1.047–1.244)	.003
Metabolic comorbidities	0.936 (0.824–1.064)	.314
Musculoskeletal comorbidities	0.916 (0.805–1.041)	.177
Gastrointestinal comorbidities	1.155 (1.026–1.300)	.017
Baseline use of ICS‐ containing medication	1.152 (1.043–1.272)	.005

*Note*: Statistically significant (*p* < .05).

Abbreviations: AED, Accident and Emergency Department; ICS, inhaled corticosteroids.

## 4 DISCUSSION

This large, multicentre, territory‐wide retrospective study evaluated the relationships between patients' demographic, clinical characteristics, baseline disease management, level of asthma control, and severe exacerbation requiring hospitalization. Similar to other large asthma studies, our cohort had a predominance of elderly, female, nonsmokers and a high prevalence of asthma‐associated comorbidities.^8^, ^17^, ^18^, ^19^, ^20^, ^21^ Increasing age, hospitalizations and AED visits for asthma exacerbation in the preceding year, presence of neuropsychiatric and gastrointestinal comorbidities and use of ICS‐containing medications at baseline were associated with an increasing number of hospitalizations for asthma exacerbation.

Ageing and asthma‐related comorbidities increase the risk of severe asthma exacerbation and healthcare costs.^11^, ^21^, ^22^ The relatively high prevalence of comorbidities may be contributed by an older age range and inclusion of an extended list of comorbidities in our study. Among the eight categories of comorbidity included in our study, neuropsychiatric and gastrointestinal comorbidities remained significantly associated with MA after adjustment of other factors (including age), thus confirming the findings from previous studies.^11^, ^21^ There is a cumulative effect of urgent healthcare utilization for asthma exacerbation (hospitalization and AED attendance) in the preceding year on the number of hospitalizations in the subsequent year. Our findings thus accord with previous reports that a history of recent healthcare utilization is the strongest predictor of future asthma exacerbations,^12^, ^18^ and extend this to include exacerbations that require hospitalizations. There is emerging evidence that this increased risk of future asthma exacerbations may last more than 5 years, although the timing of these future exacerbations is largely unpredictable.^23^ Nevertheless, a history of asthma exacerbation within a year, and probably for even a longer time frame, should flag for optimization of asthma care, especially in the elderly with neuropsychiatric and/or gastrointestinal comorbidities.

The difference between SA and MA patients in our study also shared some other similarities with previously reported cohorts, while retaining its unique features. MA patients had significantly lower pre‐ and postbronchodilator spirometric parameters than SA patients.^13^, ^24^ More MA patients had their asthma partly controlled or uncontrolled compared to the SA patients.^25^, ^26^ The recently reported association between blood eosinophils count greater than 0.4 × 10^9^/L and more severe exacerbations, however, could not be confirmed by our study.^14^ Most of our patients were elderly and might have a preponderance of T2‐low asthma. Further prospective studies to define the endotypes and phenotypes of these hospitalized patients can provide more information about their relationship with asthma exacerbations.

The level of asthma control captured in our cohort fell far short of the goals for long‐term management in international guidelines. Although it has long been recognized in community‐based studies worldwide,^27^, ^28^, ^29^, ^30^ similar data from hospitalized asthma patients, however, are lacking. Controller medications, especially ICS, were underused in our hospitalized patients with only 64.8% were receiving ICS before their hospitalizations in the current study. Even amongst those with MA, 15.6% of them were not on any controller treatment. The actual rate of using ICS‐containing regimen by asthma patients in real‐life could even be lower due to non‐adherence and poor drug inhalation technique, which are common problems among asthma patients.^31^ Moreover, workup on asthma, including lung function assessment & adequate atopic phenotyping, even amongst those with MA, was infrequent. Self‐management action plan has been proposed as an integral part of asthma management but, similar to other studies, only 10% of our patients had an action plan discussed.^16^, ^28^, ^32^, ^33^ We believe that recurrent asthma exacerbations can be reduced by reinforcing these essential components of asthma care.

Asthma care should be optimized by overcoming management gaps for several groups of asthma patients. Patients on GINA Steps 4 and 5 treatment and had multiple hospitalizations for asthma exacerbations (313, 60.3%), and those with either partly controlled or uncontrolled asthma before the index admission (616, 60.5% of those with symptom assessment documented), should have early and regular review as an important part of their usual care. Optimal care should include a proper assessment of asthma control, identify and prevent the triggers of asthma exacerbations, review diagnosis and medications adherence, and suitable pharmacological treatment. Treatment with biologics in appropriately selected patients should be considered as they have been shown to reduce exacerbations, including those required AED visit and hospitalization, and maintain disease control.^34^ It is disappointing to note that phenotyping, a prerequisite of biologic treatment, was often incomplete as only 2.6% of the whole cohort and 5.8% of patients with MAs had their atopic status assessed. However, clinical care should not only be focused on patients with severe or uncontrolled asthma. In our cohort, 38.1% of patients had seemingly controlled or mild asthma (on GINA Steps 1 or 2 treatments) before their index admissions were subsequently hospitalized at least once for their asthma exacerbation, a finding concords with other studies.^23^, ^35^, ^36^, ^37^ Over‐rating asthma control and underuse of controller medications are common features amongst asthma patients worldwide.^27^, ^28^ Physicians should actively question the presence of asthma symptoms, frequency of rescue bronchodilator usage and assess the level of asthma control during consultations as they were associated with urgent healthcare utilization.^38^ Better management strategy, including early identification of these patients and initiation of appropriate asthma treatments (with regular or as‐needed ICS),^35^ may alter disastrous clinical outcomes and prevent hospitalization.^39^ The occurrence of severe asthma exacerbation in patients without long‐term follow‐up is also worrisome. These patients were younger, with fewer comorbidities and had better spirometric parameters. They, as well as their attending physicians, might have ignored the need for follow‐up and controller medications given the apparently “mild” disease and better premorbid status. Patient education, along with a regular follow‐up, should be offered to this group of patient.

The biggest strength of this multi‐center territory‐wide study is that it comprised a large patient population from 11 participating general hospitals which covered 70% of all emergency admissions to the public healthcare sector in Hong Kong^40^; thus these patients were highly representative of the general population. The clinical diagnosis of asthma exacerbation and medication records were retrieved and verified manually by respiratory specialists, that undoubtedly improved the data accuracy. Such manual review also uncovered the real‐world practice of suboptimal asthma care. A detailed review of the demographic and clinical characteristics on this large sample of hospitalized patients with asthma exacerbations is rarely seen in other studies based on computer database search.

There are several limitations related to the study design. The retrospective study has its intrinsic disadvantage in understanding the complex interaction between asthma exacerbation and different clinical factors, including drug adherence^31^ and the exact level of asthma control, as these might not be accurately documented. COPD was not excluded in the current study and it might mimic the symptoms of an asthma exacerbation. However, the presence of COPD only contributed to a small proportion in the whole cohort (119, 5.2%), and these patients may belong to the subgroup of asthma‐COPD overlap. An arbitrary period of 12 months from January to December in the Year 2016 may fail to include hospitalization episodes that happened just before or after the Year 2016 and potentially misclassified MA patients as SA patients. Lastly, the workup for asthma and asthma‐related hospitalizations in the private sector could not be reliably evaluated through the public hospital system. However, the effect from such is likely to be small, as only 5.2% of patients in our study received medical care in the private sector and more than 90% of the population in Hong Kong seek in‐patient care in the public hospitals.

In conclusion, we examined the baseline clinical characteristics of asthma patients and identified risk factors associated with multiple hospitalizations for asthma exacerbation in this territory‐wide retrospective review. Patients with increasing age, an increasing number of hospitalizations or AED visits for asthma exacerbation in the preceding year, the presence of neuropsychiatric or GI comorbidities, and the use of ICS at the baseline were associated with an increasing number of hospitalizations due to asthma exacerbation. We have also found significant management deficiencies before their index admission for severe asthma exacerbations. Targeting these at‐risk patients with appropriate management strategy including pharmacologic (ICS‐containing medications and biologics) and nonpharmacologic (proper assessments and education) measures may reduce the risk of severe exacerbations and in turn the economic burden of asthma.

## CONFLICT OF INTERESTS

The authors declare that there are no conflict of interests.

## AUTHOR CONTRIBUTIONS

Ka Pang Chan had full access to all of the data in the study and takes responsibility for the integrity of the data and the accuracy of the data analysis, including and especially any adverse effects.

Fanny Wai San Ko, Kwun Cheung Ling, Pik Shan Cheung, Lee Veronica Chan, Yu Hong Chan, Yi Tat Lo, Chun Kong Ng, Macy Mei‐sze Lui, Kwok Sang Wilson Yee, Cee Zhung Steven Tseng, Pak Yiu Tse, Mo Lin Maureen Wong, Kah Lin Choo, Wai Kei Lam, Chun Man Wong, Sheng Sheng Ho, Chung Tat Lun, and Christopher Kei Wai Lai contributed substantially to the study design, data analysis and interpretation, and the writing of the manuscript.

5

## Data Availability

Research data are not shared.

## References

[1] GBD 2015 Chronic Respiratory Disease Collaborators . Global, regional, and national deaths, prevalence, disability‐adjusted life years, and years lived with disability for chronic obstructive pulmonary disease and asthma, 1990‐2015: a systematic analysis for the Global Burden of Disease Study 2015. Lancet Respir Med. 2017;5(9):691‐706. 28822787 10.1016/S2213-2600(17)30293-X PMC5573769

[2] Lai CK , Beasley R , Crane J , Foliaki S , Shah J , Weiland S , International Study of Asthma Allergies in Childhood Phase Three Study Group . Global variation in the prevalence and severity of asthma symptoms: phase three of the International Study of Asthma and Allergies in Childhood (ISAAC). Thorax. 2009;64(6):476‐483. 19237391 10.1136/thx.2008.106609

[3] Huang K , Yang T , Xu J , et al. Prevalence, risk factors, and management of asthma in China: a national cross‐sectional study. Lancet. 2019;394(10196):407‐418. 31230828 10.1016/S0140-6736(19)31147-X

[4] Loftus PA , Wise SK . Epidemiology of asthma. Curr Opin Otolaryngol Head Neck Surg. 2016;24(3):245‐249. 26977741 10.1097/MOO.0000000000000262

[5] Braman SS . Asthma in the elderly. Clin Geriatr Med. 2017;33(4):523‐537. 28991648 10.1016/j.cger.2017.06.005

[6] O'Byrne PM , Pedersen S , Lamm CJ , Tan WC , Busse WW , START Investigators Group . Severe exacerbations and decline in lung function in asthma. Am J Respir Crit Care Med. 2009;179(1):19‐24. 18990678 10.1164/rccm.200807-1126OC

[7] Luskin AT , Chipps BE , Rasouliyan L , Miller DP , Haselkorn T , Dorenbaum A . Impact of asthma exacerbations and asthma triggers on asthma‐related quality of life in patients with severe or difficult‐to‐treat asthma. J Allergy Clin Immunol Pract. 2014;2(5):544‐552e1‐2. 25213047 10.1016/j.jaip.2014.02.011

[8] Krishnan V , Diette GB , Rand CS , et al. Mortality in patients hospitalized for asthma exacerbations in the United States. Am J Respir Crit Care Med. 2006;174(6):633‐638. 16778163 10.1164/rccm.200601-007OC PMC2648055

[9] Suruki RY , Daugherty JB , Boudiaf N , Albers FC . The frequency of asthma exacerbations and healthcare utilization in patients with asthma from the UK and USA. BMC Pulm Med. 2017;17(1):74. 28449686 10.1186/s12890-017-0409-3 PMC5406966

[10] Lai CKW , Kim YY , Kuo S‐H , Spencer M , Williams AE . Cost of asthma in the Asia‐Pacific region. Eur Respir Rev. 2006;15:10‐16.

[11] Porsbjerg C , Menzies‐Gow A . Co‐morbidities in severe asthma: clinical impact and management. Respirology. 2017;22(4):651‐661. 28328160 10.1111/resp.13026

[12] Miller MK , Lee JH , Miller DP , Wenzel SE , TENOR Study Group . Recent asthma exacerbations: a key predictor of future exacerbations. Respir Med. 2007;101(3):481‐489. 16914299 10.1016/j.rmed.2006.07.005

[13] Kitch BT , Paltiel AD , Kuntz KM , et al. A single measure of FEV1 is associated with risk of asthma attacks in long‐term follow‐up. Chest. 2004;126(6):1875‐1882. 15596687 10.1378/chest.126.6.1875

[14] Price DB , Rigazio A , Campbell JD , et al. Blood eosinophil count and prospective annual asthma disease burden: a UK cohort study. Lancet Respir Med. 2015;3(11):849‐858. 26493938 10.1016/S2213-2600(15)00367-7

[15] Reddel HK , Taylor DR , Bateman ED , et al. An official American Thoracic Society/European Respiratory Society statement: asthma control and exacerbations: standardizing endpoints for clinical asthma trials and clinical practice. Am J Respir Crit Care Med. 2009;180(1):59‐99. 19535666 10.1164/rccm.200801-060ST

[16] GINA . 2016 GINA Report, Global Strategy for Asthma Management and Prevention. Global Initiation for Asthma. 2016.

[17] Tsai CL , Lee WY , Hanania NA , Camargo CA Jr. . Age‐related differences in clinical outcomes for acute asthma in the United States, 2006‐2008. J Allergy Clin Immunol. 2012;129(5):1252‐1258 e1. 22385630 10.1016/j.jaci.2012.01.061

[18] Kang HR , Song HJ , Nam JH , et al. Risk factors of asthma exacerbation based on asthma severity: a nationwide population‐based observational study in South Korea. BMJ Open. 2018;8(3):e020825. 10.1136/bmjopen-2017-020825 PMC5875610 29567854

[19] Lin J , Xing B , Tang H , et al. Hospitalization due to asthma exacerbation: a China Asthma Research Network (CARN) retrospective study in 29 provinces across mainland China. Allergy Asthma Immunol Res. 2020;12(3):485‐495. 32141261 10.4168/aair.2020.12.3.485 PMC7061152

[20] Kaur BP , Lahewala S , Arora S , et al. Asthma: hospitalization trends and predictors of in‐hospital mortality and hospitalization costs in the USA (2001‐2010). Int Arch Allergy Immunol. 2015;168(2):71‐78. 26595589 10.1159/000441687

[21] Chen W , Safari A , FitzGerald JM , Sin DD , Tavakoli H , Sadatsafavi M . Economic burden of multimorbidity in patients with severe asthma: a 20‐year population‐based study. Thorax. 2019;74(12):1113‐1119. 31534029 10.1136/thoraxjnl-2019-213223

[22] Bloom CI , Nissen F , Douglas IJ , Smeeth L , Cullinan P , Quint JK . Exacerbation risk and characterisation of the UK's asthma population from infants to old age. Thorax. 2018;73(4):313‐320. 29074814 10.1136/thoraxjnl-2017-210650

[23] Bloom CI , Palmer T , Feary J , Quint JK , Cullinan P . Exacerbation patterns in adults with asthma in England. A population‐based study. Am J Respir Crit Care Med. 2019;199(4):446‐453. 30507307 10.1164/rccm.201808-1516OC

[24] Koga T , Oshita Y , Kamimura T , Koga H , Aizawa H . Characterisation of patients with frequent exacerbation of asthma. Respir Med. 2006;100(2):273‐278. 15998585 10.1016/j.rmed.2005.05.017

[25] Chipps BE , Szefler SJ , Simons FER , et al. Demographic and clinical characteristics of children and adolescents with severe or difficult‐to‐treat asthma. J Allergy Clin Immunol. 2007;119(5):1156‐1163. 17397912 10.1016/j.jaci.2006.12.668

[26] Fleming L . Asthma exacerbation prediction: recent insights. Curr Opin Allergy Clin Immunol. 2018;18(2):117‐123. 29406359 10.1097/ACI.0000000000000428

[27] Lai CKW , De Guia TS , Kim YY , et al. Asthma control in the Asia‐Pacific region: the asthma insights and reality in Asia‐Pacific study. J Allergy Clin Immunol. 2003;111(2):263‐268. 12589343 10.1067/mai.2003.30

[28] Rabe KF , Adachi M , Lai CKW , et al. Worldwide severity and control of asthma in children and adults: the global asthma insights and reality surveys. J Allergy Clin Immunol. 2004;114(1):40‐47. 15241342 10.1016/j.jaci.2004.04.042

[29] Thompson PJ , Salvi S , Lin J , et al. Insights, attitudes and perceptions about asthma and its treatment: findings from a multinational survey of patients from 8 Asia‐Pacific countries and Hong Kong. Respirology. 2013;18(6):957‐967. 23730953 10.1111/resp.12137

[30] Price D , Fletcher M , van der Molen T . Asthma control and management in 8,000 European patients: the REcognise Asthma and LInk to Symptoms and Experience (REALISE) survey. NPJ Prim Care Respir Med. 2014;24:14009. 24921985 10.1038/npjpcrm.2014.9 PMC4373302

[31] Engelkes M , Janssens HM , de Jongste JC , Sturkenboom MC , Verhamme KM . Medication adherence and the risk of severe asthma exacerbations: a systematic review. Eur Respir J. 2015;45(2):396‐407. 25323234 10.1183/09031936.00075614

[32] Pinnock H . Supported self‐management for asthma. Breathe (Sheff). 2015;11(2):98‐109. 26306110 10.1183/20734735.015614 PMC4487370

[33] Cloutier MM , Salo PM , Akinbami LJ , et al. Clinician agreement, self‐efficacy, and adherence with the guidelines for the diagnosis and management of asthma. J Allergy Clin Immunol Pract. 2018;6(3):886‐894 e4. 29408439 10.1016/j.jaip.2018.01.018 PMC5948143

[34] McGregor MC , Krings JG , Nair P , Castro M . Role of Biologics in Asthma. Am J Respir Crit Care Med. 2019;199(4):433‐445. 30525902 10.1164/rccm.201810-1944CI PMC6835092

[35] Beasley R , Holliday M , Reddel HK , et al. Controlled trial of budesonide‐formoterol as needed for mild asthma. N Engl J Med. 2019;380(21):2020‐2030. 31112386 10.1056/NEJMoa1901963

[36] Romagnoli M , Caramori G , Braccioni F , et al. Near‐fatal asthma phenotype in the ENFUMOSA Cohort. Clin Exp Allergy. 2007;37(4):552‐557. 17430352 10.1111/j.1365-2222.2007.02683.x

[37] Restrepo RD , Peters J . Near‐fatal asthma: recognition and management. Curr Opin Pulm Med. 2008;14(1):13‐23. 18043271 10.1097/MCP.0b013e3282f1982d

[38] Lai CKW , Ko FWS , Bhome A , et al. Relationship between asthma control status, the Asthma Control Test and urgent health‐care utilization in Asia. Respirology. 2011;16(4):688‐697. 21362102 10.1111/j.1440-1843.2011.01954.x

[39] Suissa S , Ernst P , Benayoun S , Baltzan M , Cai B . Low‐dose inhaled corticosteroids and the prevention of death from asthma. N Engl J Med. 2000;343(5):332‐336. 10922423 10.1056/NEJM200008033430504

[40] Hospital Authority Annual Report 2016‐2017 *Available at*: https://www.ha.org.hk/ho/corpcomm/AR201617/ebook/en/mobile/index.html#p=I. *Assessed July 7 2020*.

